# Genetic analyses of the plasma proteome and metabolome from the same cohort pinpoint AD risk associated molecular phenotypes

**DOI:** 10.1002/alz.085269

**Published:** 2025-01-03

**Authors:** Chengran Yang, Priyanka Gorijala, Jigyasha Timsina, Lihua Wang, Ciyang Wang, Menghan Liu, David M. Holtzman, John C. Morris, Yun Ju Sung, Carlos Cruchaga

**Affiliations:** ^1^ Washington University, Saint Louis, MO USA; ^2^ Washington University School of Medicine, Saint Louis, MO USA; ^3^ NeuroGenomics & Informatics Center, Washington University School of Medicine, St. Louis, MO USA; ^4^ Department of Psychiatry, Washington University School of Medicine, St. Louis, MO USA; ^5^ Washington University in St. Louis, School of Medicine, St. Louis, MO USA

## Abstract

**Background:**

The recent European‐ancestry based genome‐wide association study (GWAS) of Alzheimer disease (AD) by Bellenguez2022 has identified 75 significant genetic loci, but only a few have been functionally mapped to effector gene level. Besides the large‐scale RNA expression, protein and metabolite levels are key molecular traits bridging the genetic variants to AD risk, and thus we decided to integrate them into the genetic analysis to pinpoint key proteins and metabolites underlying AD etiology. Few studies have generated more than one layer of post‐transcriptional phenotypes, limiting the scale of biological translation of disease modifying treatments.

**Methods:**

We first performed the plasma proteomic (6,907 proteins by SomaScan) and metabolomic (1,483 metabolites by Metabolon) GWAS from the same participants of European (N = 2,300) ancestry. Using these significant variant‐trait associations, we next performed multiple post‐GWAS analyses (functional summary‐based imputation (FUSION) and genetic colocalization) to identify the AD associated proteins and metabolites. To annotate these findings with brain aging clocks predicated from the plasma proteomics data, we performed association tests between the aging gaps with the molecular phenotypes.

**Results:**

We identified hundreds to thousands of QTLs (quantitative trait loci). In the proteomic GWAS, we found 2,400 proteins with 2,848 study‐wide significant pQTLs. In the metabolomic GWAS, we reported 403 metabolites with 490 study‐wide significant mQTLs. In total, 86% and 98% of these associations have supported by the previous larger‐scale plasma‐based studies in proteome and metabolome, respectively. Under the FUSION framework, we found 95 proteins were study‐wide significant associated with AD risk, including, proteins TREM2, APOE, and NfL. After removing the bias of linkage disequilibrium by colocalization, we obtained a list of 53 proteins, of which 42 were not reported. On the other hand, 35 nominal significant metabolites, such as androsterone sulfate, were associated with AD in the FUSION analysis. Of these findings, eight metabolites were also highly colocalized with AD. There were 14 proteins and three metabolites significantly associated with the brain aging gaps, which included TREM2, but not APOE.

**Conclusions:**

Our study serves the first duo‐omics post‐transcriptional genetic study for studying AD risk effectors and can facilitate developing novel disease modifying treatments.

